# Integrative stemness characteristics associated with prognosis and the immune microenvironment in lung adenocarcinoma

**DOI:** 10.1186/s12890-022-02184-8

**Published:** 2022-12-05

**Authors:** Han Wang, Ying Wang, Wei Luo, Xugang Zhang, Ran Cao, Zhi Yang, Jin Duan, Kun Wang

**Affiliations:** 1 Department of Thoracic Surgery, The First People’s Hospital of Yunnan Province, The Affiliated Hospital of Kunming University of Science and Technology, 650031 Kunming, Yunnan China; 2 Department of Thoracic Surgery, Yan’an Hospital of Kunming, 650000 Kunming, Yunnan China; 3 Department of Thoracic Surgery, The Affiliated Anning First People’s Hospital, Kunming University of Science and Technology, Kunming Fourth People’s Hospital, No. 2 Ganghe Road, Wanghu Neighborhood Committee, Jinfang Street, 650302 Anning, Yunnan China; 4 The IVD Medical Marketing Department, 3D Medicines Inc, 201114 Shanghai, China; 5 Department of Thoracic Surgery, the First Affiliated Hospital of Kunming Medical University, 650031 Kunming, Yunman China

**Keywords:** Lung adenocarcinoma, Tumor microenvironent, Prognosis, Stemness, mRNAsi

## Abstract

**Background:**

To comprehensively analyze the stemness characteristics related to prognosis and the immune microenvironment in lung adenocarcinoma (LUAD).

**Methods:**

The OCLR machine learning method was used to calculate the stemness index (mRNAsi) of the LUAD samples. DEGs common between the low mRNAsi, normal, and high mRNAsi groups were screened and the immune-stemness genes were obtained. Then the PPI network was created and enrichment analyses were performed. Moreover, different subtypes based on immune-stemness genes associated with prognosis were identified, and the relationships between LUAD stemness and TIME variables were systematically analyzed, followed by TMB analysis.

**Results:**

Patients in the high mRNAsi groups with poor prognosis were screened along with 144 immune-stemness genes. IL-6, FPR2, and RLN3 showed a higher degree in the PPI network. A total of 26 immune-stemness genes associated with prognosis were screened. Two clusters were obtained (cluster 1 and cluster 2). Survival analysis revealed that patients in cluster 2 had a poor prognosis. A total of 12 immune cell subpopulations exhibited significant differences between cluster 1 and cluster 2 (*P* < 0.05). A total of 10 immune checkpoint genes exhibited significantly higher expression in cluster 1 (*P* < 0.05) than in cluster 2. Further, the TMB value in cluster 2 was higher than that in cluster 1 (*P* < 0.05).

**Conclusion:**

Immune-stemness genes, including L-6, FPR2, and RLN3, might play significant roles in LUAD development via cytokine–cytokine receptor interaction, neuroactive ligand‒receptor interaction, and the JAK‒STAT pathway. Immune-stemness genes were related to tumor-infiltrating immune cells, TMB, and expression of immune checkpoint gene.

**Supplementary Information:**

The online version contains supplementary material available at 10.1186/s12890-022-02184-8.

## Highlights

- IL-6, FPR2, RLN3 had higher degree in PPI network.
- The patients in cluster 1 had better prognosis.
- Total 10 immune checkpoint genes showed higher expression in cluster 1.
- TMB value in cluster 2 was higher than that in cluster 1.

**Supplementary Information:**

The online version contains supplementary material available at 10.1186/s12890-022-02184-8.

## Background

Lung cancer is a malignant disease with the highest incidence rate and mortality rate across the world [1, 2]. Lung adenocarcinoma (LUAD) is a major type of lung cancer, accounting for 40–50% of all lung cancers and originates from the bronchial epithelial and mucous glands [3, 4]. Despite significant improvements in targeted and chemotherapeutic techniques, the overall survival rate of patients with LUAD is still poor [5]. Therefore, it is essential to screen markers related to LUAD prognosis.

Cancer stem cells (CSCs) have the ability of self-renewal and producing heterogeneous tumor cells [6]. However, owing to their biological characteristics and the protective effect of the tumor microenvironment (TME), CSCs are less sensitive to conventional radiotherapy and chemotherapy, a phenomenon that supports tumor recurrence and metastasis [7]. Therefore, if CSCs are not completely removed, relapse and metastasis can easily occur. Transcriptional and epigenetic disorders of cancer cells often alter the core signaling pathways—regulating the phenotype of normal stem cells—leading to carcinogenic dedifferentiation and acquisition of stemness [8]. In addition, the complex interactions between immune cells and their secreted molecules in the TME help maintain the viability and self-renewal ability of the CSCs [9]. The stemness index (mRNAsi) is an index used to describe the similarity between tumor cells and stem cells. Higher mRNAsi is related to active biological process and a higher degree of tumor dedifferentiation [10]. LUAD comprises a complex system of cancer cells, infiltrating immune cells, CSCs, and nonmalignant stromal cells. However, a synthesized understanding of the tumor immune microenvironment (TIME) and LUAD stemness is lacking.

In this study, an OCLR machine learning method was used to calculate the mRNAsi of the LUAD samples. Then, the samples were divided into low and high mRNAsi groups based on the median value of mRNAsi. The DEGs between the low mRNAsi group vs. normal group, high mRNAsi group vs. normal group, and high mRNAsi group vs. low mRNAsi group were screened, and immune-stemness genes were obtained. PPI network analysis and enrichment analysis were performed. Moreover, different subtypes based on immune-stemness genes associated with prognosis were acquired. The relationships between LUAD stemness and TIME variables were systematically evaluated, followed by tumor mutation burden (TMB) analysis. This study offers novel understanding of the stemness characteristics of LUAD and provides a theoretical basis for drug development.

## Methods

### Data source and data preprocessing

**Fig. 1:**
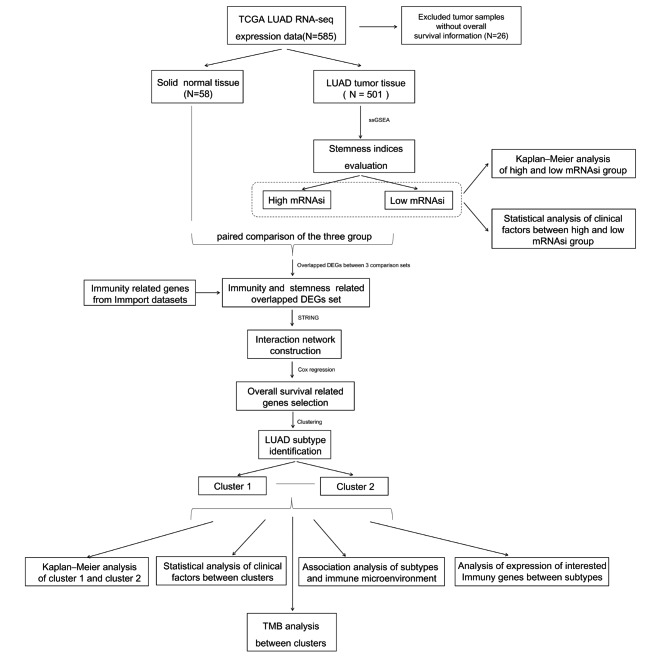
Flow chart of this study

The gene expression data of 585 LUAD patients (collected using an Illumina HiSeq 2000 RNA Sequencing platform-based) were acquired from TCGA. In total, 501 LUAD tumor samples and 58 normal samples were obtained after retaining LUAD samples and normal samples with survival prognosis information. Ethical approval was obtained for accessing patient information curated on the database, and all methods were carried out in accordance with relevant application guidelines and regulations of TCGA. This study is based on open source data, so there are no ethical issues. A flowchart of this study is shown in Fig. 1.

### Evaluating the clinical significance and prognosis of mRNAsi

The mRNAsi of LUAD samples was calculated using OCLR machine learning [11] through the gelnet package in R software. Thereafter, the differences in mRNAsi values between LUAD samples and normal samples were evaluated using a *t*-test. In addition, the LUAD samples were categorized into low and high mRNAsi groups based on the median value of mRNAsi, and Kaplan‒Meier survival analysis was used to assess the prognosis of patients between the low and high mRNAsi groups. Fisher’s exact test (count variables such as sex) and *t*-test (continuous variables such as age) were used to assess the differences in clinical information between the low and high mRNAsi groups.

### PPI network and enrichment analysis of immune-stemness genes

The samples were allocated into normal sample, low mRNAsi, and high mRNAsi sample groups based on the source information and mRNAsi grouping information of the samples. Thereafter, the limma package [12] of R was used to identify the DEGs between the low mRNAsi group vs. normal group, high mRNAsi group vs. normal group, and high mRNAsi group vs. low mRNAsi group with the following cutoffs: false positive discovery (FDR) < 0.05; |log_2_FC| > 0.5. Common DEGs were then obtained. In addition, immune-related genes were acquired from the Immport database [13]. The common DEGs were intersected with these immune-related genes and the overlapping genes were redefined as immune-stemness genes.

STRING [14] was used to analyze the interactions between immune-stemness genes encoding proteins with a PPI score > 0.7. Cytoscape [15] was used to build the PPI network. Moreover, enrichment analysis of immune-stemness genes in the PPI network was performed using DAVID [16, 17] with an FDR threshold < 0.05.

### Identification of different subtypes based on immune-stemness genes related to prognosis

Immune-stemness genes in the PPI network were subjected to univariate Cox regression analysis using the survival package [18] to screen the immune-stemness genes significantly associated with survival prognosis with a threshold of *P* < 0.05. In addition, based on the expression of immune-stemness genes related to prognosis, the pheatmap package [19] in R software was used to conduct bidirectional hierarchical cluster analysis to identify the different subtypes using the centered Pearson correlation algorithm [20]. Subsequently, Kaplan‒Meier survival analysis was used to assess the prognosis of patients between these different subtypes. Fisher’s exact test (count variables such as sex) and *t*-test (continuous variables such as age) were used to evaluate the differences in clinical information between these different subtypes.

### Association between different subtypes with TIME

Based on the expression profile of LUAD samples, CIBERSORT [21] was used to evaluate 22 types of tumor-infiltrating immune cells in these subtypes. The differences in the expression of numerous immune checkpoint genes were compared.

### TMB analysis of different subtypes

As the biology of LUAD is different depending on the type of oncogenic driver mutations, the relations between driver mutations [Kirsten rat sarcoma viral oncogene homolog (KRAS), epidermal growth factor receptor **(**EGFR), anaplastic lymphoma kinase (ALK), c-Ros oncogene 1 receptor tyrosine kinase (ROS1), V-raf murine sarcoma viral oncogene homolog B1 (BRAF), mesenchymal-epithelial transition factor (MET), and RET proto-oncogene (RET)] and the stemness score were compared. In addition, based on the LUAD gene mutation data obtained from TCGA, the gene mutation frequencies of LUAD samples were evaluated. *t*-test was used to assess the differences in gene mutation frequency between these different subtypes. TMB is generally defined as the number of somatic coding mutations per million bases. The TMB of LUAD samples was calculated using the maftools package [22] in R, and the differences in TMB between these different subtypes were compared using Wilcox test.

### Statistical analysis

Fisher’s exact test was used to compare the differences in count variables between groups. *t*-test was used to compare the differences in continuous variables between groups, and the normalization was conducted before using the *t*-test, and non-parametric methods was used if the parameters were not normally distributed. *P* < 0.05 was considered significant.

## Results

### Evaluating the clinical significance and prognosis of mRNAsi

**Fig. 2:**
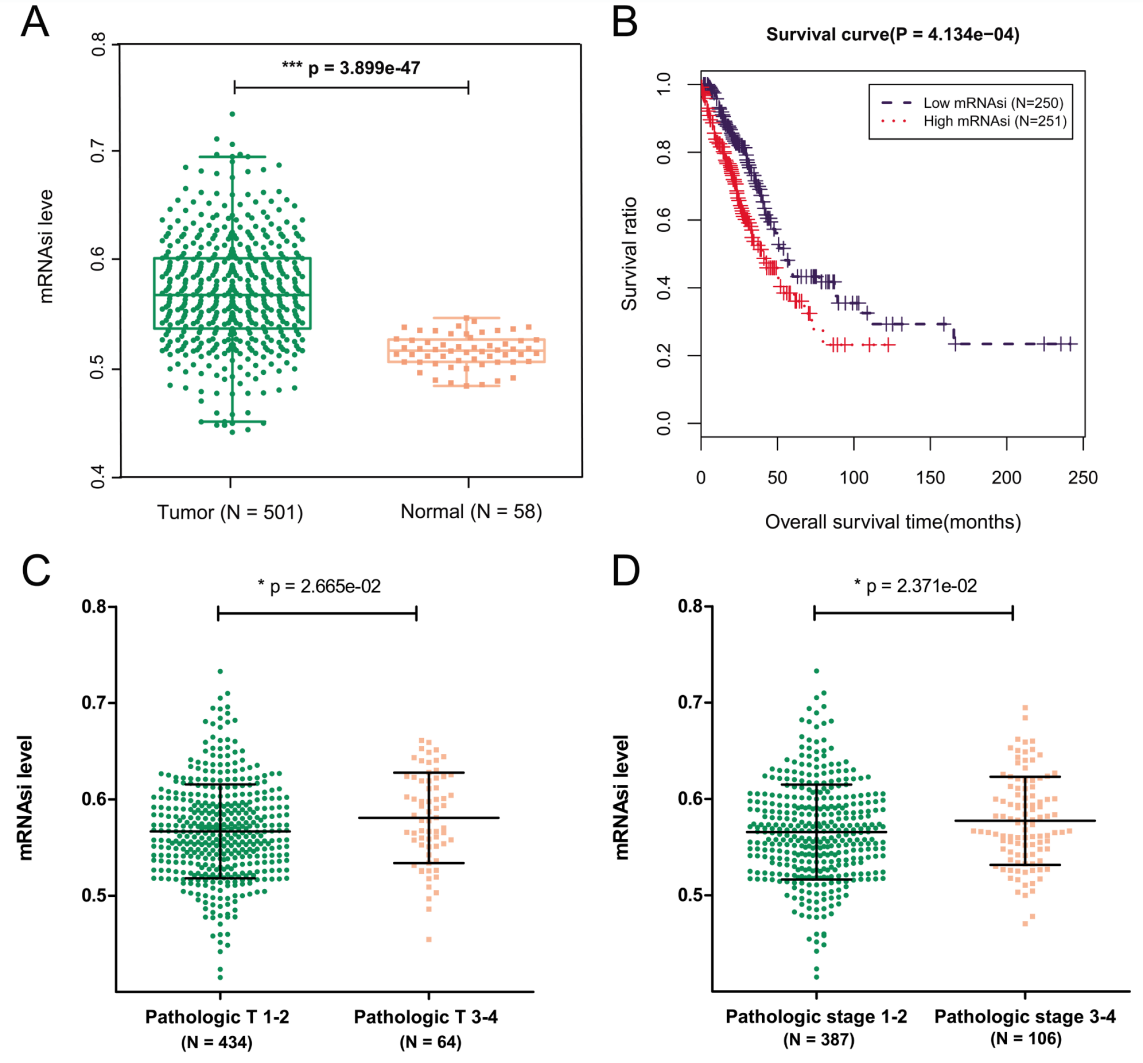
Evaluation of the clinical significance and prognosis of mRNAsi. **(A)** The mRNAsi value corresponding to LUAD samples and normal samples. **(B)** Survival analysis of low and high mRNAsi groups. Difference in the pathological T **(C)** and pathological stage **(D)** between the low and high mRNAsi groups

As shown in Fig. 2 A, the mRNAsi value corresponding to that of LUAD samples was higher than that corresponding to that of normal samples. Survival analysis revealed that the patients in the high mRNAsi group had a poor prognosis (Fig. 2B). In addition, differences in clinical information between the low and high mRNAsi groups were examined (Table 1). The results revealed that pathological T (Fig. 2 C) and pathological stage (Fig. 2D) were significantly different between the low and high mRNAsi groups (*P* < 0.05), and the mRNAsi value was positively correlated with pathological T and stage, which explains the poor prognosis in the high mRNAsi group.

**Table 1:** Statistics and comparative analysis of clinical information in high and low mRNAsi groups

characteristics total cases	N of case 501	mRNAsi level		P value
		**Low**	**High**	
Age(years)				
≤ 60	157	86	71	1.493E-01
> 60	344	164	180	
Geneder				
Male	231	115	116	9.986E-01
Female	270	135	135	
Pathologic M				
M0	333	165	168	6.791E-01
M1	24	13	11	
Pathologic N				
N0	324	163	161	7.819E-01
N1	94	46	48	
N2	72	39	33	
Pathologic T				
T1	167	79	88	**2.060E-02**
T2	267	143	124	
T3	45	21	24	
T4	19	6	13	
Pathologic stage				
Stage I	268	148	120	**3.337E-03**
Stage II	119	62	57	
Stage III	81	26	55	
Stage IV	25	13	12	
Tumor recurrence				
Yes	74	77	151	9.309E-01
No	131	144	275	
Radiotherapy				
Yes	32	28	60	8.093E-01
No	194	194	388	

### PPI network enrichment analysis of immune-stemness genes

**Fig. 3:**
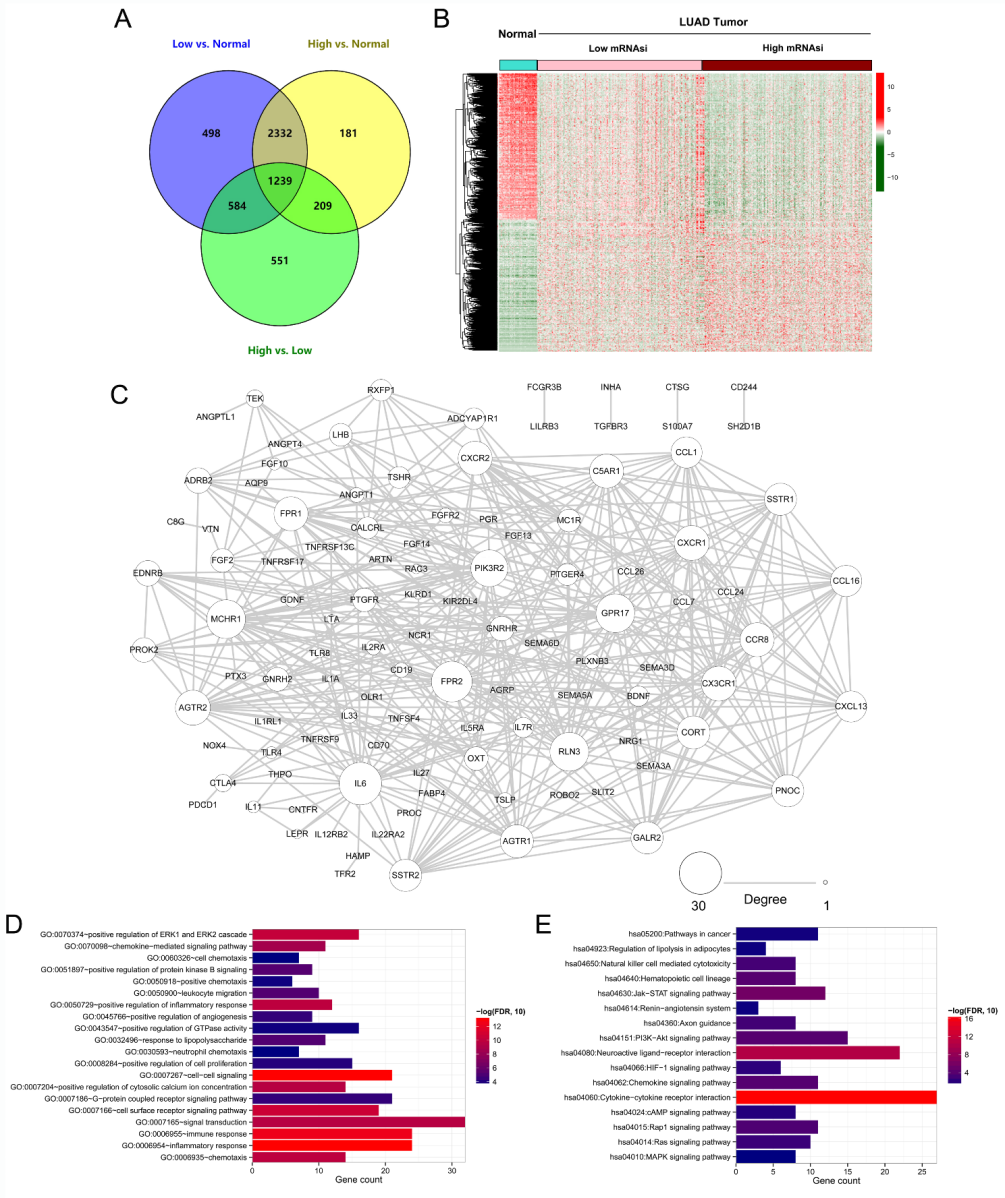
Protein-protein interaction (PPI) network and enrichment analysis of immune-stemness genes. **(A)** Differentially expressed genes (DEGs) and common DEGs screened from low mRNAsi group vs. normal group, high mRNAsi group vs. normal group and high mRNAsi group vs. low mRNAsi group. **(B)** The expression level of common DEGs in samples from the high mRNAsi and low mRNAsi groups. **(C)** PPI network of these immune-stemness genes; the large nodes with higher degree. Gene ontology (GO) **(D)** and Kyoto Encyclopedia of Genes and Genomes (KEGG) pathway analysis **(E)** of the immune-stemness genes in the PPI network

As previously mentioned, a total of 4653, 3961, and 2583 DEGs were screened from the low mRNAsi group vs. normal group, high mRNAsi group vs. normal group, and high mRNAsi group vs. low mRNAsi group, respectively, and a total of 1239 common DEGs were identified (Fig. 3 A). The expression levels of common DEGs were significantly different between samples from the high and low mRNAsi groups (Fig. 3B). In addition, 144 immune-stemness genes were identified. The PPI network of these immune-stemness genes showed that there were 109 nodes in the PPI network (Fig. 3 C), and interleukin 6 (IL-6), formyl peptide receptor-2 (FPR2), and relaxin 3 (RLN3) had a higher degree in the PPI network (Supplementary Table ); thus, L-6, FPR2, and RLN3 might play significant roles in LUAD development. Subsequently, enrichment analysis was carried out on the immune-stemness genes in the PPI network. The results showed that the 109 immune-stemness genes were involved in 42 biological processes (BPs) (Fig. 3D) and 16 Kyoto Encyclopedia of Genes and Genomes (KEGG) pathways (Fig. 3E) [23, 24].

### Identification of different subtypes based on immune-stemness genes associated with prognosis

**Fig. 4:**
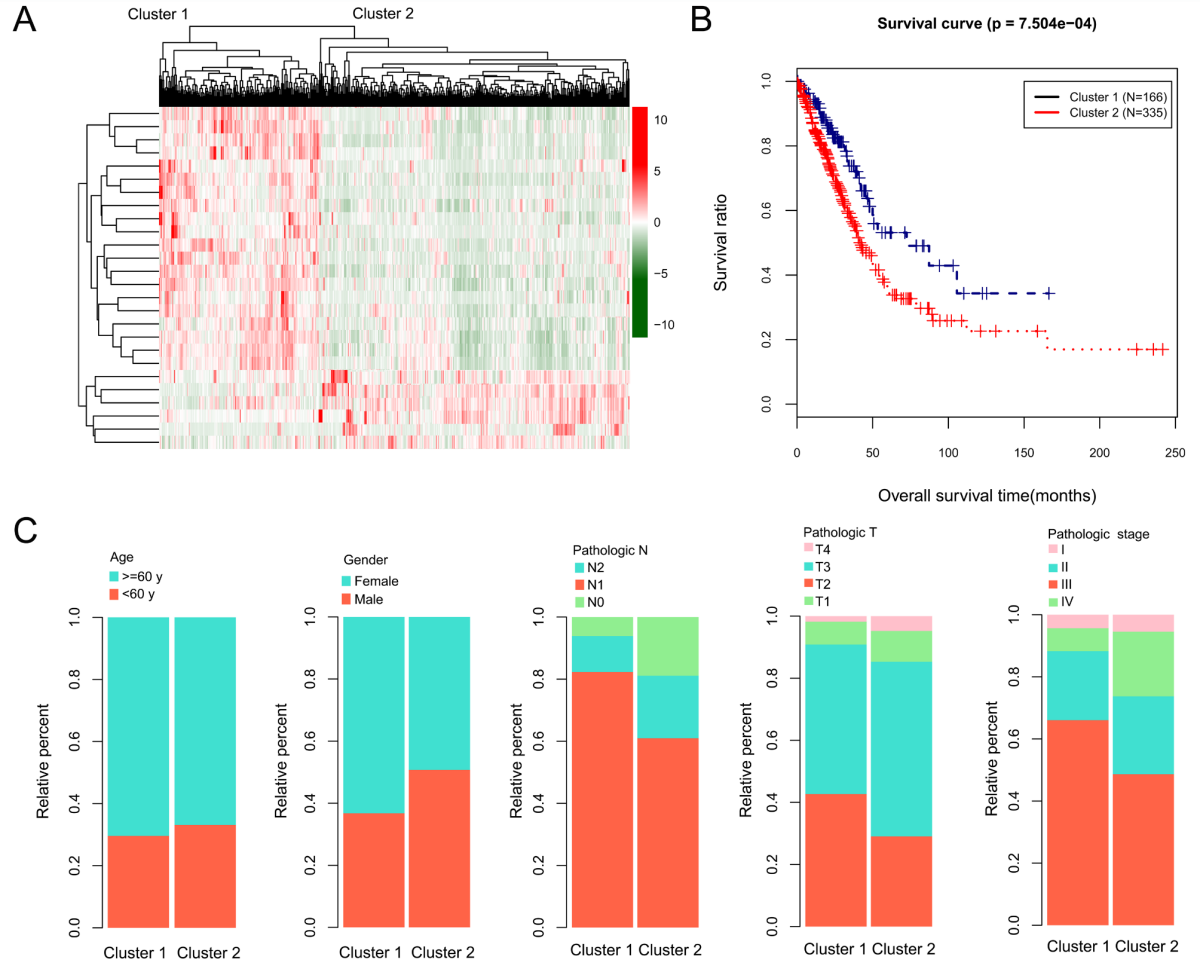
Identification of different subtypes based on immune-stemness genes associated with prognosis. **(A)** Identification of the different subtypes. **(B)** Survival analysis cluster 1 and cluster 2. **(C)** Differences in age, sex, pathologic N, pathologic T, and pathologic stage between cluster 1 and cluster 2

A total of 26 immune-stemness genes associated with prognosis were screened (Supplementary Table 2). Two clusters were identified, cluster 1 and cluster 2; there were 166 and 335 samples in cluster 1 and cluster 2, respectively (Fig. 4 A). Survival analysis showed that the patients in cluster 2 had a poor prognosis (Fig. 4B). The differences in age, sex, pathologic N, pathologic T, and pathologic stage between cluster 1 and cluster 2 were significant (*P* < 0.05) (Fig. 4 C and Table 2).

**Table 2:** Statistics and comparison of clinical information of samples in different subtype clusters using Wilcox test

Clinical characteristics	TCGA (N = 501)	Cluster 1 (N = 166)	Cluster 2 (N = 335)	P value
Age(years, mean ± sd)	65.28 ± 10.05	66.79 ± 10.36	64.53 ± 9.822	0.02
Gender(Male/Female)	231/270	61/105	170/165	3.208e-03
Pathologic M(M0/M1/-)	333/24/144	106/6/54	227/18/90	6.496e-01
Pathologic N(N0/N1/N2/-)	324/94/72/11	121/27/9/9	203/67/63/2	8.045e-05
Pathologic T(T1/T2/T3/T4/-)	167/267/45/19/3	70/79/12/3/2	97/188/33/16/1	1.439e-02
Pathologic stage( I / II / III / IV /-)	268/119/81/25/8	107/36/12/7/4	161/83/69/18/4	1.775e-04
Tumor recurrence(Yes/No/-)	151/275/75	45/107/14	106/168/61	7.218e-02
Radiotherapy(Yes/No/- )	60/388/53	16/136/14	44/252/39	2.417e-01

### Association between different subtypes with TIME

**Fig. 5:**
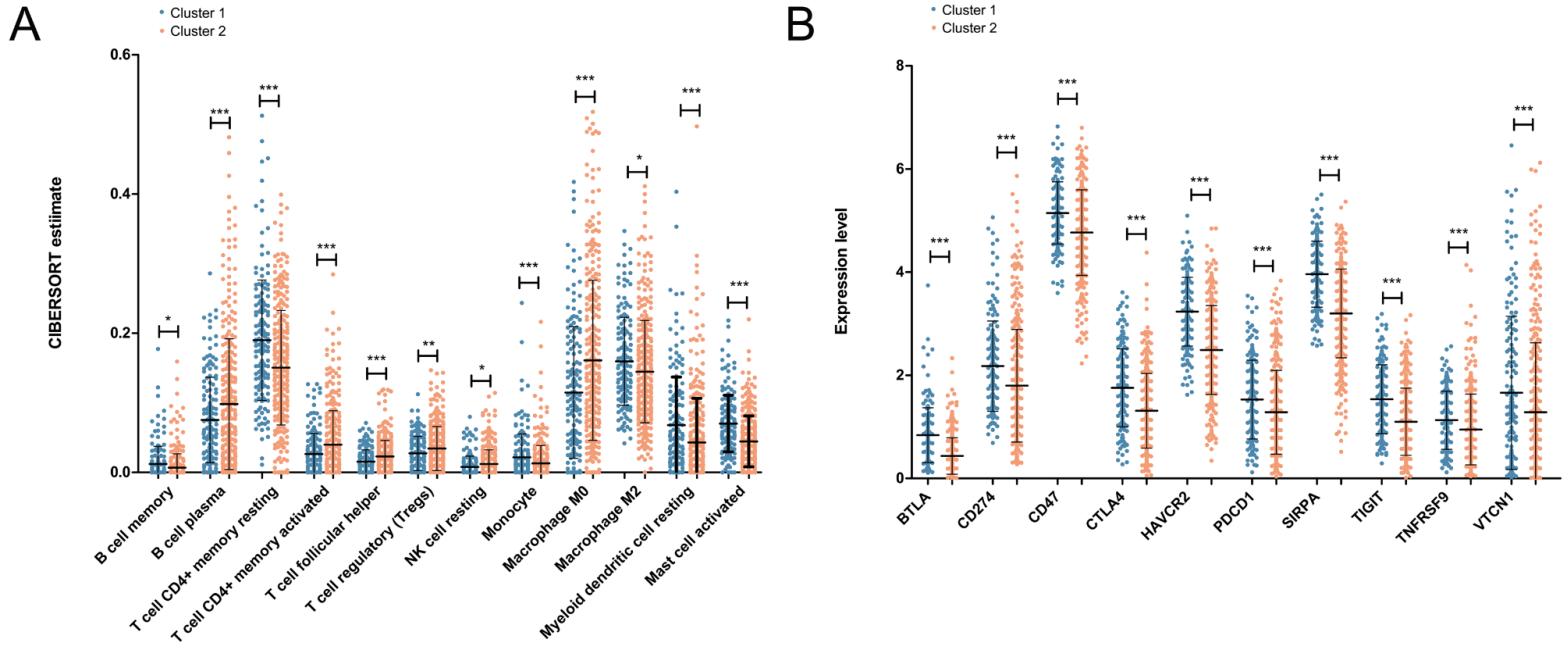
Association between different subtypes with tumor immune microenvironment (TIME). **(A)** Differences in immune cell subpopulations between the cluster 1 and cluster 2 groups. **(B)** Differences in the expression levels of immune checkpoint genes between cluster 1 and cluster 2 groups. * represents *P* < 0.05

A total of 22 infiltrating immune subpopulations were evaluated; among them, 12 immune cell subpopulations showed significant differences between cluster 1 and cluster 2 (*P* < 0.05) (Fig. 5 A). A comparison of the differences in expression levels of immune checkpoint genes between cluster 1 and cluster 2 revealed that 10 immune checkpoint genes showed significantly higher expression in cluster 1 than in cluster 2 (*P* < 0.05) (Fig. 5B). These results provided new insights into LUAD development and immunotherapy.

### TMB analysis of different subtypes

**Fig. 6:**
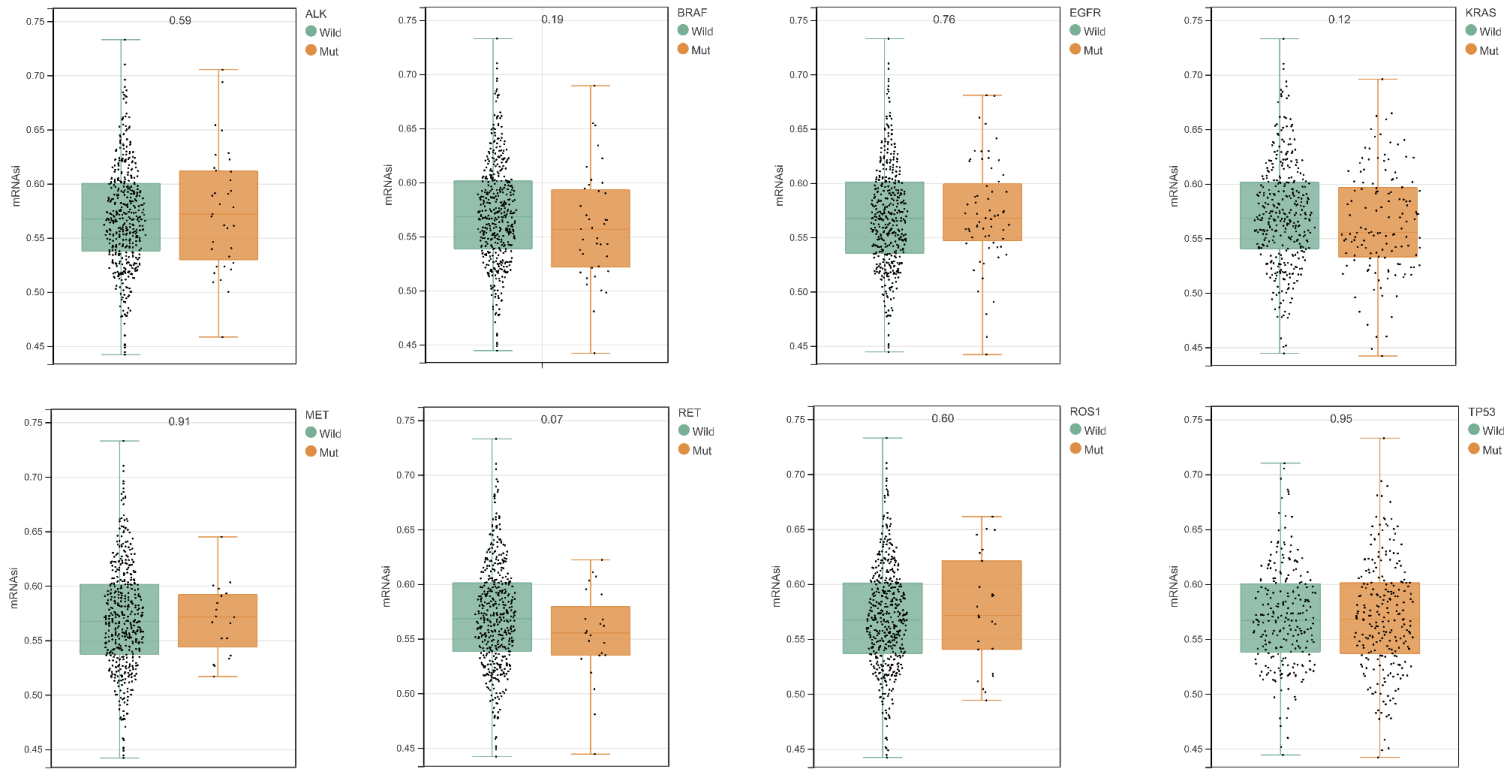
Association between driver mutations and the stemness score

**Fig. 7:**
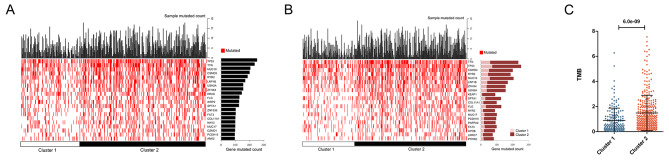
Tumor mutation burden (TMB) analysis of different subtypes. **(A)** Gene mutation frequency of LUAD samples. **(B)** Gene mutation frequency in cluster 1 and cluster 2. **(C)** TMB value in cluster 1 and cluster 2

As shown in Fig. 6, the stemness scores of KRAS, EGFR, ALK, ROS1, BRAF, MET, and RET showed no significant difference between the Mut and Wild groups (P > 0.05). The gene mutation frequency of LUAD samples was determined; tumor protein p53 (TP53), titin (TTN), mucin 16, cell surface associated (MUC16), etc. had a high mutation frequency (Fig. 7 A); the mutation frequency of TP53, TTN, CUB, and Sushi multiple domains 3 (CSMD3), among others, in cluster 1 and cluster 2 were significantly different (Fig. 7B). The TMB value in cluster 2 was higher than that in cluster 1 (*P* < 0.05) (Fig. 7 C), which is explained by the results of survival analysis.

## Discussion

In this study, patients in the high mRNAsi group with a poor prognosis and a total of 144 immune-stemness genes were screened. There were 109 nodes in the PPI network, and enrichment analysis revealed that the 109 immune-stemness genes were involved in 42 BPs and 16 KEGG pathways. In total, 26 immune-stemness genes associated with prognosis were screened. Two clusters were identified, namely, cluster 1 and cluster 2. Survival analysis revealed that patients in cluster 2 had a poor prognosis. A total of 12 immune cell subpopulations showed significant differences between cluster 1 and cluster 2 (*P* < 0.05), and a total of 10 immune checkpoint genes showed significantly higher expression in cluster 1 than in cluster 2 (*P* < 0.05); TMB values in cluster 2 were higher than those in cluster 1.

Studies have suggested that mRNAsi might serve as an effective index for the survival, classification, and disease progression of tumor patients [25–27]. Huang et al. found that basal breast cancer patients have high mRNAsi values [28]. In the present study, we found that the mRNAsi value in LUAD samples was higher than that in normal samples, and survival analysis revealed that patients in the high mRNAsi group had a poor prognosis. These findings are in accordance with the results of the above studies. In addition, clinical analysis showed that the mRNAsi value was positively correlated with pathological T and stage, and patients with higher pathological T and stage had a higher mRNAsi value. These findings verify the results of survival analysis.

A total of 144 immune-stemness genes were screened in this study; IL-6, FPR2, and RLN3 had a high degree in the PPI network and therefore could be considered the hub genes. IL-6 is a pleiotropic four-helix bundle cytokine that plays various functions in the body [29]. The IL-6 family is one of the most important cytokine families involved in the process of tumorigenesis and metastasis, particularly IL-6 [30]. IL-6 promotes tumorigenesis by regulating all hallmarks of cancer and multiple signaling pathways [31]. FPR2 is a G-protein coupled receptor that plays a major role in cancer development and inflammation [32]. Several studies have shown that FPR2 stimulates tumor cell invasion and proliferation [33, 34]. For instance, Lu et al. showed that FPR2 enhances colorectal cancer progression by promoting epithelial–mesenchymal transition process [35]. Xie et al. found that FPR2 is involved in epithelial ovarian cancer progression through RhoA-mediated M2 macrophage polarization [36]. In addition, Zhang et al. found that RLN3 may be a key gene affecting the progression of hepatocellular carcinoma [37]. Liu et al. revealed that RLN3 was differentially expressed and prognostically relevant in patients with KRAS-mutant colorectal cancer [38]. KEGG pathway analysis revealed that the 109 immune-stemness genes in the PPI network were involved in 16 KEGG pathways, including cytokine−receptor interaction, neuroactive ligand−receptor interaction, and JAK−STAT signaling pathway. Cytokine receptor interaction may be the key to determining the role of inflammation in disease development [39]. The JAK−STAT signaling pathway is highly associated with many inflammatory and immune diseases [40]. Zhou et al. found that cytokine−receptor interaction and JAK−STAT signaling pathway are related to the development of glioblastoma [41]. Chen et al. showed that neuroactive ligand−receptor interaction is correlated with the occurrence of glioma [42], suggesting that these immune-stemness genes might play key roles in LUAD development.

Immunotherapy has become an effective treatment for cancer; immune cells are an important part of the TME and play vital roles in tumor immunotherapy [43]. In this study, two clusters were screened, including cluster 1 and cluster 2, and the survival analysis uncovered that the patients in cluster 1 had a better prognosis. A total of 12 immune cell subpopulations, including M0 macrophages, regulatory T cells (Tregs), and memory B cells, showed significant differences between cluster 1 and cluster 2. A total of 10 immune checkpoint genes, including PDCD1, CTLA4, and CD274, showed significantly higher expression in cluster 1 than in cluster 2. Among immune checkpoint therapies, PD-1/PD-L1 and CTLA-4 inhibitors have shown promising therapeutic outcomes [44]. TMB is a new biomarker for predicting the effect of immunotherapy [45, 46]. Negrao et al. showed that low TMB was a predictive factor for worse outcomes in lung cancer [47]. Zhang et al. found that high TMB levels led to poor survival outcomes in clear cell renal cell carcinoma [48]. In this study, we found that the TMB value in cluster 2 was higher than that in cluster 1, which explains why cluster 1 had a better prognosis.

This study has some limitations. First, the data analyzed in this study were all publicly available; thus, the key genes and molecular mechanisms should be explored in further experiments in the future. Second, the infiltrating immune subpopulations were evaluated using only the CIBERSORT algorithm; therefore, further tools and relevant experiments should be carried out to validate our findings. Third, large-scale prospective clinical studies are required to evaluate the obtained immune-stemness genes.

## Conclusion

Immune-stemness genes, including L-6, FPR2, and RLN3, might play significant roles in LUAD development via cytokine–cytokine receptor interaction, neuroactive ligand–receptor interaction, and JAK–STAT signaling pathway. In addition, immune-stemness genes were related to tumor-infiltrating immune cells, TMB, and immune checkpoint gene expression. Therefore, this study proposed novel insights into the clinical treatment of LUAD.

## Electronic supplementary material

Below is the link to the electronic supplementary material.

Supplementary Material 1

## Data Availability

The datasets used and/or analyzed during the current study are available from TCGA (dataset ID: TCGA-LUAD.htseq_fpkm.tsv; https://gdc-hub.s3.us-east-1.amazonaws.com/download/TCGA-LUAD.htseq_fpkm.tsv.gz; Full metadata).

## List of abbreviations

**LUAD**: Lung adenocarcinoma
**CSCs**: Cancer stem cells
**TME**: Tumor microenvironment
**TIME**: Tumor immune microenvironment
**TMB**: Tumor mutation burden
**FPR2**: Formyl peptide receptor-2
**RLN3**: Relaxin 3
**BPs**: Biological processes
**TP53**: Tumor protein p53
**TTN**: Titin
**MUC16**: Mucin 16
**Tregs**: Regulatory T cells
