# Supplementary material for: Integrative stemness characteristics associated with prognosis and the immune microenvironment in lung adenocarcinoma
Source: BMC Pulm Med. 2022 Dec 5;22:463. doi: 10.1186/s12890-022-02184-8 (PMC9724367; doi:10.1186/s12890-022-02184-8)
Supplement: Supplementary file 1 — Supplementary Material 1 [file 12890_2022_2184_MOESM1_ESM.doc]

Supplementary table 1 The degree of 109 nodes in the PPI network (top 10).

| ID | AverageShortestPathLength | BetweennessCentrality | ClosenessCentrality | Degree |
| --- | --- | --- | --- | --- |
| IL6 | 2.04 | 0.41214223 | 0.49019608 | 30 |
| FPR2 | 2.29 | 0.06234782 | 0.43668122 | 28 |
| RLN3 | 2.57 | 0.08093823 | 0.38910506 | 26 |
| GPR17 | 2.31 | 0.03619294 | 0.43290043 | 26 |
| MCHR1 | 2.31 | 0.03619294 | 0.43290043 | 26 |
| PIK3R2 | 2.25 | 0.27845937 | 0.44444444 | 24 |
| AGTR2 | 2.61 | 0.00545716 | 0.38314176 | 22 |
| CX3CR1 | 2.31 | 0.04381083 | 0.43290043 | 22 |
| CXCR1 | 2.31 | 0.04381083 | 0.43290043 | 22 |
| CXCR2 | 2.65 | 0.00751317 | 0.37735849 | 21 |

Supplementary table 2 Immune-stemness genes associated with prognosis.

| Symbol | coef | exp(coef) | se(coef) | z | p |
| --- | --- | --- | --- | --- | --- |
| IL1A | 0.245 | 1.28 | 0.0733 | 3.34 | 0.00084 |
| ADRB2 | -0.369 | 0.692 | 0.112 | -3.3 | 0.00097 |
| CX3CR1 | -0.348 | 0.706 | 0.106 | -3.28 | 0.001 |
| INHA | 0.121 | 1.13 | 0.0397 | 3.05 | 0.0023 |
| CTSG | -0.338 | 0.713 | 0.116 | -2.92 | 0.0035 |
| CD19 | -0.251 | 0.778 | 0.0881 | -2.85 | 0.0043 |
| IL11 | 0.304 | 1.36 | 0.111 | 2.74 | 0.0061 |
| RXFP1 | -0.967 | 0.38 | 0.355 | -2.72 | 0.0065 |
| IL33 | -0.172 | 0.842 | 0.0642 | -2.68 | 0.0075 |
| TNFRSF17 | -0.186 | 0.83 | 0.0703 | -2.65 | 0.008 |
| ROBO2 | -0.492 | 0.611 | 0.187 | -2.64 | 0.0084 |
| IL5RA | -0.576 | 0.562 | 0.226 | -2.55 | 0.011 |
| FGFR2 | -0.211 | 0.81 | 0.0841 | -2.51 | 0.012 |
| TSLP | -1.1 | 0.333 | 0.435 | -2.53 | 0.012 |
| ANGPT1 | -0.265 | 0.767 | 0.106 | -2.49 | 0.013 |
| SEMA3A | 0.156 | 1.17 | 0.063 | 2.48 | 0.013 |
| IL7R | -0.156 | 0.856 | 0.0651 | -2.39 | 0.017 |
| CTLA4 | -0.237 | 0.789 | 0.1 | -2.36 | 0.018 |
| TEK | -0.249 | 0.779 | 0.107 | -2.32 | 0.02 |
| TNFRSF13C | -0.229 | 0.795 | 0.0994 | -2.31 | 0.021 |
| VTN | 0.183 | 1.2 | 0.0803 | 2.28 | 0.023 |
| MC1R | 0.308 | 1.36 | 0.141 | 2.18 | 0.029 |
| FGF14 | -0.591 | 0.554 | 0.279 | -2.12 | 0.034 |
| TLR8 | -0.21 | 0.811 | 0.101 | -2.08 | 0.038 |
| LILRB3 | -0.313 | 0.731 | 0.152 | -2.06 | 0.039 |
| TLR4 | -0.176 | 0.839 | 0.0882 | -1.99 | 0.046 |
